# Preparation of a Colorimetric TLC Stain for Amines from Furfural

**DOI:** 10.1021/acs.jchemed.6c00213

**Published:** 2026-06-30

**Authors:** João R. Vale, Carlos A. M. Afonso, Rafael F. A. Gomes

**Affiliations:** Research Institute for Medicines (iMed.ULisboa), Faculty of Pharmacy, 70880 Universidade de Lisboa, Avenida Professor Gama Pinto, 1649-003, Lisbon, Portugal

**Keywords:** Second-Year Undergraduate, Organic Chemistry, Hands-On Learning, Amines, Green Chemistry

## Abstract

A laboratory experiment was developed for the preparation of a thin-layer chromatography stain from the condensation of furfural and *N*-methylbarbituric acid. The experiment allows for the introduction of the concepts of selective TLC staining and colorimetric detection of amines. The stain is based on the formation of donor–acceptor-Stenhouse-adducts (DASAs), and its preparation and application allow the introduction of organic chemistry concepts such as the Knoevenagel condensation, ring opening, and Nazarov electrocyclization, as well as photochromic systems. The cheap reagents required for this work and easy setup allow for a straightforward introduction of the work in various settings, including academic laboratories. The use of biomass-derived furfural reinforces the importance of green chemistry and sustainable practices in organic chemistry.

## Graphical abstract

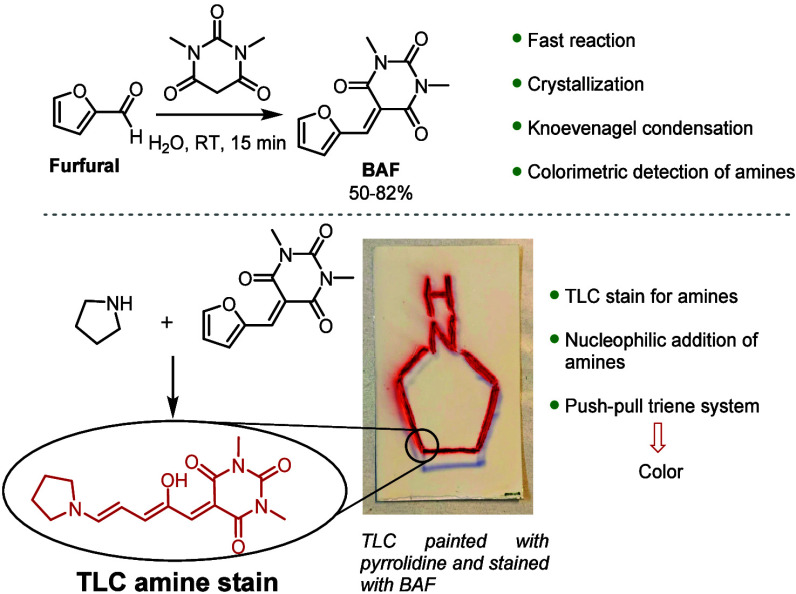

Amines are one of the major functional groups that are present in pharmaceuticals and several important chemicals.^1–4^ For this reason, it is a fundamental functional group and methodologies for their preparation and reactivity are taught in most organic chemistry curricula. A topic often overlooked is colorimetric methodologies for the detection of amines.^5–11^ Indeed, most methods rely on spectroscopy or other techniques such as HPLC or GC, with ninhydrin appearing as the most common TLC stain for amines.^12–16^ Taking advantage of a known photochromic system, the donor–acceptor-Stenhouse-adducts (DASAs),^17–27^ Alaniz and co-workers have previously reported the detection of amines using activated furfurals such as the barbituric acid-furan adduct (BAF) and the Meldrum’s acid-furan adduct (MAF) as depicted in Scheme 1.^28^

**Scheme 1:**
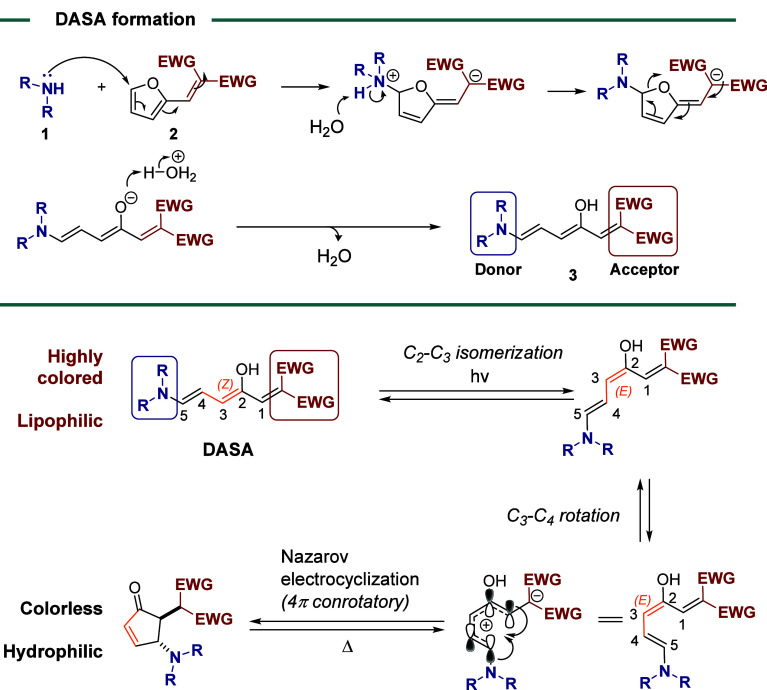
Addition of an Amine to Activated Furans to form DASAs (Top) and Their Rearrangement to Cyclopentenones (Bottom)

These furfural-based photochromic switches have been the subject of several research and review articles, mostly because of their visible light absorption spectra and tunable photochromism.

DASAs emerge from the concept of Stenhouse salts, originally discovered by J. Stenhouse in 1850 from the condensation of aniline with furfural rich crude oil obtained from plants.^29,30^ These are highly colored trienes which can undergo thermal 4π-electrocyclization^31^ to yield colorless 4,5-diamino-2-cyclopentenones, which further rearrange into the thermodynamically more stable 2,4-isomers. These remained underexplored until the early 2000s when Batey and co-workers discovered that Lewis acids could promote the selective transformation of furfural to 4,5-diamino-2-cyclopentenones under mild conditions.^32^ Further improvements on the technology led to greener and water compatible conditions described by Procopio, Nardi and us.^33–35^ Despite their importance as scaffolds and synthons for total synthesis of natural products, these Stenhouse salts were transient intermediaries that led to the formation of colorless cyclopentenones, thus not being ideal for the detection of amines. To this end, Alaniz in 2014 described the activation of furfural with carbon acids, such as Meldrum’s acid and *N*-methyl-barbituric acid to yield MAF and BAF.^26,27^ These activated furans undergo fast reaction with secondary amines yielding stable Stenhouse adducts, which due to the donor nature of the amine and acceptor nature of the carbon acid moiety were named donor–acceptor-Stenhouse-adducts. The authors observed that the DASAs behave as photochromic systems, losing their color when irradiated due to the Nazarov electrocyclization to the corresponding colorless cyclopentenone. Importantly, when heated in the appropriate solvent the cyclopentenone can undergo thermic reversion to the colored DASA. Several groups have been involved in various applications of these photochromic systems, ranging from material to drug release applications.^18,23,24,36,37^

Their facile preparation and accessibility of the reagents make DASAs ideal candidates for undergraduate students to learn chemistry laboratory techniques, as well as several theoretical fundamentals of organic chemistry. In particular: (i) reactivity of aldehydes, which undergoes Knoevenagel condensation with a β-dicarbonyl compounds; (ii) electrocyclization, namely the Nazarov electrocyclization;^38–40^ (iii) colorimetric detection of functional groups.

## Pedagogic Goals

This experiment has two main focuses: the first being the reactivity principles behind a Knoevenagel condensation and the second being the colorimetric detection of amines by the formation of a highly colored product. The use of furfural as starting reagent and water as the solvent media allows the instructor to further discuss green chemistry principles and introduce furfural as a biorenewable building block.^41^ Furfural is a mass produced bulk chemical obtained from lignocellulosic material, with a market size reaching 370 K t/y which allows further discussion on sustainable chemistry.^42,43^

The undergraduate organic chemistry students will also learn carbonyl reactivity, particularly aldehydes and their condensation and addition reactions with nucleophiles, which is a topic covered in organic chemistry courses. In fact, other reports on pedagogic experiments involving Knoevenagel condensation usually require harsher conditions, both high temperature and catalyst, whereas this one is performed at room temperature and catalyst-free. This allows discussion of the acidity of barbituric acids and how this improves the reactivity toward aldehydes.^44,45^ The students will also tackle amine nucleophilicity and evaluate differences in the reactivity of different nitrogen bases.

## Hazards

The use of protective clothing, gloves, and protection goggles is mandatory, such as common good laboratory practices (GLP). Handling waste and chemicals is done according to the safety data sheets (SDS). There are no halogenated waste concerns, however, the heterogeneous catalysts should be disposed of properly with a protection mask to protect students from inhaling silica. Furfural is toxic if swallowed or inhaled, harmful in the case of skin contact (irritant) or eye contact (irritant), and may cause respiratory irritation. Ethyl acetate is a highly flammable liquid and vapor, may cause drowsiness or dizziness, and causes serious eye irritation. Petroleum ether is a highly flammable liquid and vapor, may be fatal if swallowed, and enters airways, causes skin irritation, and may cause drowsiness or dizziness. Silica should be handled with care because of the risk of chronic silicosis with prolonged inhalation.

## Laboratory Experiment

The experiment was carried out by 14 undergraduate students, distributed over two sessions, during the second year organic chemistry II course of the Integrated Master Course of Pharmaceutical Sciences at the Faculty of Pharmacy, University of Lisbon. This laboratory experiment was aligned with the course curriculum regarding the “reactivity of the carbonyl group” and “amine functional group” topics.^46^ It was further carried out by 7 high school students as a first contact with university laboratories, in a program to introduce the faculty to local high school students.

The experiment was performed in under 3 h, starting from the preparation of the activated furan BAF, followed by the characterization of the products and colorimetric TLC staining. It is worth noting that both high school students with little experience and the undergraduate students with previous organic laboratory experience achieved similar results (see the Supporting Information (SI) Table S1).

Initially it was planned for the students to prepare two different furfural adducts, one with barbituric acid-furan and another with Meldrum’s acid. However, the experiment with Meldrum’s acid presented a very slow crystallization step that required manipulation of solvent proportions, leading to poor yields, irreproducible results, and impure products. Ultimately, we removed the preparation of MAF for further classes and focused on the barbituric derivative (Scheme 2).

**Scheme 2:**
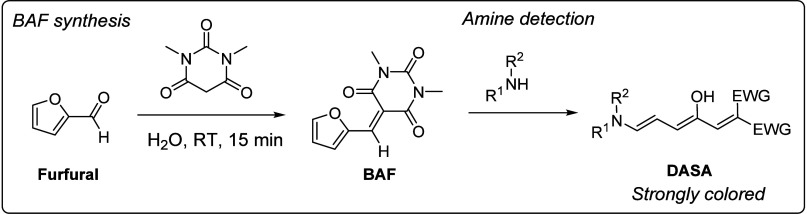
Preparation of Barbituric Acid-Furan (BAF) and Its Reaction with Amines to form DASAs

### Preparation of the Activated Furan BAF

The students start this work by preparing the activated furfural adduct with *N,N*-dimethylbarbituric acid (BAF, Scheme 2). This allows for discussion on the importance of biorenewable feedstocks, green chemistry, and the Knoevenagel reaction. The reaction was adapted from previously reported methodologies.^26^

The students stirred 276 mg (1.77 mmol) of *N,N*-dimethyl-barbituric acid in 13 mL of distilled water in a 25 mL round-bottom flask until total dissolution. Then, 136 μL (1.64 mmol) of furfural was added quickly with a gastight syringe and the mixture was allowed to stir for 15 min. The mixture was cooled down in an ice bath and the precipitate was filtered through a Büchner funnel and washed with cold water. The powder can be used as is or can be further purified by recrystallization in hot ethanol. The crystals were allowed to dry 10 min in an oven at 60 °C. The product was then characterized by measuring the melting point and ^1^H NMR. Upon analysis of the ^1^H NMR spectral data, the students were asked to identify the olefinic C–H in the benzylic position of the furan ring of BAF (8.44 ppm), which is shielded in comparison with the starting material aldehyde C–H signal (9.69 ppm).

The student teams were able to achieve moderately good yields (50–82%). A detailed list of the yields and melting points obtained from the different teams can be accessed in Table S1.

### Amine Staining

After isolating the BAF product, the students prepared six samples in a 10 mg/mL concentration of different nitrogen bases, that were selected to present differently colored DASAs after reaction with BAF:

(i) aniline
(ii) 1,2,3,4-tetrahydroquinoline (THQ)
(iii) morpholine
(iv) *N,N*-diisopropylethylamine (DIPEA)
(v) acetanilide
(vi) *n*-hexylamine

TLC plates were spotted with the six nitrogen bases (Figure 1B) and eluted with ethyl acetate:heptane (50:50). After evaluating the plate under a UV lamp (254 nm, Figure 1A), students prepared a solution of BAF (45 mg in 10 mL of ethyl acetate) and dipped the TLC plate into the solution (Figure 1C). Upon dipping, some spots become immediately colored (morpholine turns bright red and hexylamine yellow, corresponding to the most nucleophilic alkylamines). Then, the spot of THQ turns blue and aniline purple, which sometimes required some heating. Arylamines/anilines are less nucleophilic than alkyls due to resonance of the nitrogen lone pair to the aromatic ring, which is reflected in different coloration of their DASA products and the speed of their formation. Then, the spot of THQ turns blue and aniline purple, which sometimes requires some heating with a heat gun. Arylamines/anilines are less nucleophilic than alkyls due to resonance of the nitrogen lone pair to the aromatic ring, which is reflected in different coloration of their DASA products and the speed of their formation. In some cases, for DIPEA, students observed a faint blue spot. Being a tertiary amine, it is not expected to form DASA as there is no proton to abstract after amine addition in molecule **3** (Scheme 1). In addition, the bulkiness around the nitrogen atom in DIPEA decreases its nucleophilicity and should hinder its nucleophilic addition to furfuryl alcohol. It is possible that the coloration originates from secondary amine impurities present in DIPEA that are detected because of the high sensitivity of the colorimetric TLC stain. In fact, a very similar stain profile was observed for diisopropylamine (Figure 1D), a precursor to DIPEA and a possible contaminant. Acetanilide, being an amide, is not expected to be nucleophilic toward furfural as the nitrogen lone pair is in strong resonance with the carbonyl group. As such, no color was observed.

**Figure 1:**
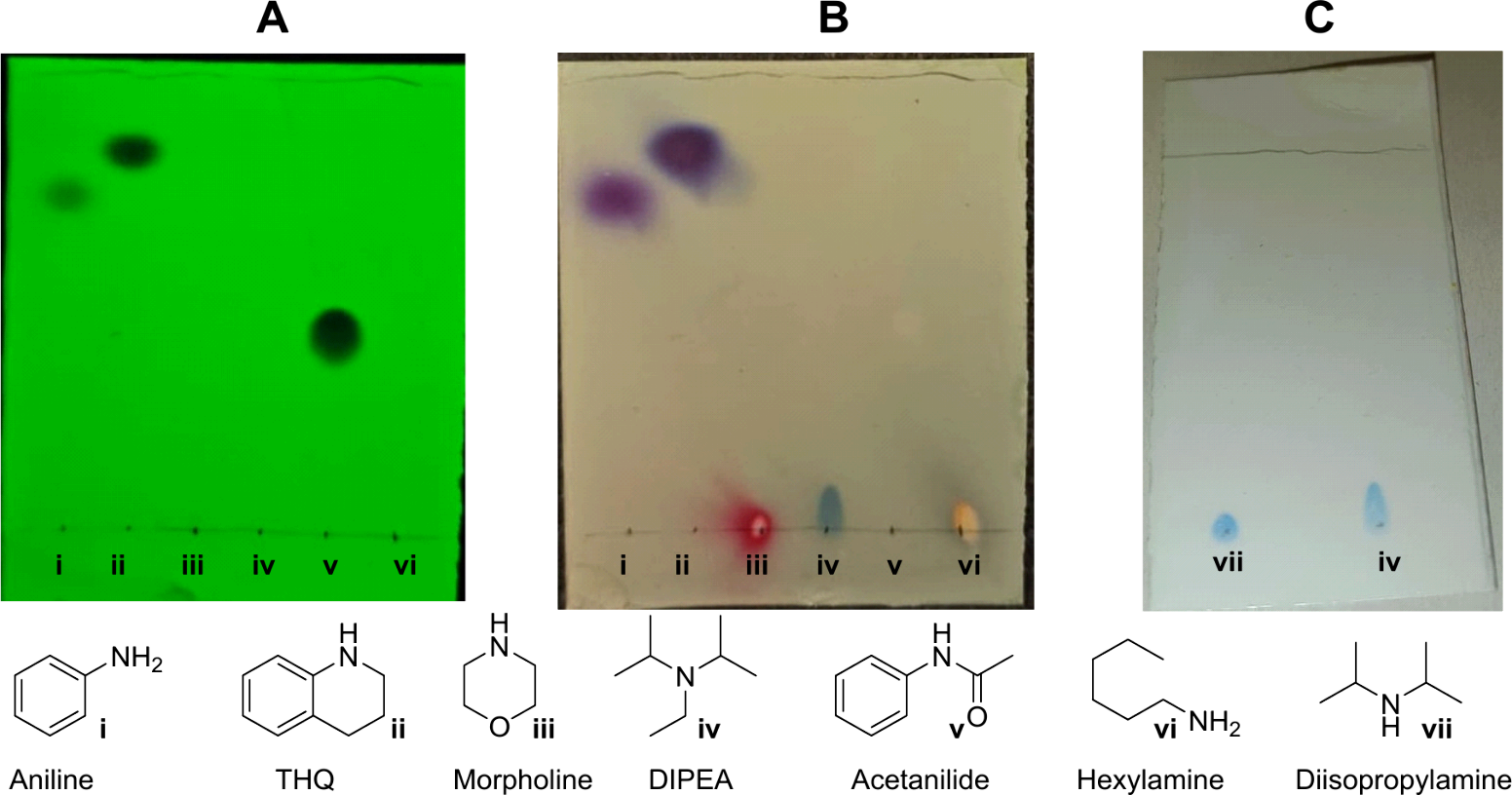
TLC plate spotted with amines **i–vi** and eluted with EtOAc:heptane (40:60) under UV light (A) and after staining with BAF (B). TLC of DIPEA and diisopropyl amine eluted and stained with BAF (C) showing similar staining profile.

Placing the TLC under UV light allows the discussion of the visibility of amines bearing aromatic motifs being detectable, whereas alkyl amines are not visible and, thus, require staining for identification.

## Assessment

Assessment of the learning outcomes was performed during the laboratory experiment and from a final quiz. The students were already proficient in common laboratory practices such as weighing, preparing solutions, evaporating solvents under reduced pressure, thin-layer chromatography, and calculating reaction yields. In fact, all students were able to calculate the yield with an average of 58% yield.

The students were asked to solve a quiz concerning the topics of reactivity of aromatic compounds, acidity/basicity, amine vs amide nucleophilicity, and NMR spectroscopy. 80% of the students were able to identify why the amine addition occurs at position 5 rather than position 4. Answers often included resonance structures of the activated furfural upon addition of the amine in position 5 and in position 4, showing increased stabilization by resonance in the former. 90% of the students drew the resonance structures of the dimethylbarbiturate to justify the high acidity of barbituric acid. All of the students were able to identify that the acetanilide was a less basic nitrogen-bearing compound due to being an amide and the lone nitrogen pair being in resonance with the carbonyl. Likewise, the students also identified aniline and tetrahydroquinoline as the less basic amines due to the conjugation of the lone pair to the aromatic system. 90% of the students correlated the absence of color of acetanilide after staining to the poor nucleophilicity of the amide, which in turn does not form the colored DASA. All of the students were able to attribute the ^1^H NMR spectra of furfural and BAF. This assessment revealed that the students learned key concepts of furan reactivity/nucleophilic addition to aromatic systems, colorimetric detection, acidity, and basicity. The instructors may discuss in increased detail the mechanism of Knoevenagel condensation for the undergraduate students who are learning the carbonyl group reactivity.

## Ethics

Regarding ethical considerations, this study did not involve the collection of identifiable personal information. All student data were collected anonymously and analyzed in an aggregate. Prior to participation, students were informed that their laboratory work and anonymized responses could be used for research and publication purposes. The study falls under a general institutional protocol for classroom activities.

## Conclusions

The preparation of barbituric acid activated furan and its use as a colorimetric TLC stain for amines was performed successfully by the undergraduate students of the Integrated Master Pharmaceutical Sciences course. The experiment allowed the introduction of specific concepts of colorimetric detection of functional groups, electrocyclization, photochromic switches and consolidate the reactivity of aldehyde and β-dicarbonyl functionalities. The purity of the crystalline products can be easily determined with a melting point apparatus, and no specific glassware is required for this experiment other than common organic classroom glassware available in a teaching laboratory of organic Chemistry. Overall, the feedback from the students was positive, and for most of them became their first contact with the aforementioned concepts.

## Supplementary Material



## Notes

^1^H and ^13^C NMR spectra for all compounds, additional experimental information (yields, melting points and R_f_s), and student handouts survey and solutions (PDF)
