# Supplementary material for: Preparation of a Colorimetric TLC Stain for Amines from Furfural
Source: J Chem Educ. 2026 Jun 30;103(7):3893–8. doi: 10.1021/acs.jchemed.6c00213 (PMC13374104; doi:10.1021/acs.jchemed.6c00213)
Supplement: Supplementary file 1 [file ed6c00213_si_001.pdf]

Preparation of a Colorimetric TLC Stain for Amines from Furfural

João R. Vale,* Carlos A. M. Afonso, Rafael F. A. Gomes*,

Research Institute for Medicines (iMed.ULisboa), Faculty of Pharmacy, Universidade de Lisboa, Avenida Professor Gama Pinto, 1649-003, Lisbon (Portugal). jvale@ff.ulisboa.pt, rafael.gomes@campus.ul.pt;

Contents

General information 1

Knoevenagel Reaction 2

Compound characterization 2

^{1}H NMR Spectra (300 MHz, $CDCl_3$) 4

^{13}C NMR Spectra (75 MHz, $CDCl_3$) 5

Thin Layer Chromatography 6

Student handouts 7

Questions and discussion (solved) 12

General information

NMR spectra were recorded in a Bruker Fourier 300 (Bruker, Massachusetts, USA) using $CDCl_3$ as deuterated solvent. Chemical shifts are given in parts per million (ppm). All coupling constants are expressed in Hz and chemical shifts (δ) in ppm. Multiplicities are given as: s (singlet), d (doublet), dd (double doublet), dt (double triplet), t (triplet), m (multiplet). TLC analysis was performed on Merck 60 F254 silica gel. Melting points were determined using a Stuart™ melting point apparatus SMP10 and were not corrected. All reagents used were purchased from BLD Fluorochem TCI or Sigma-Aldrich and used without further purification.

Knoevenagel Reaction

Table S1. Reaction yields from the different teams:

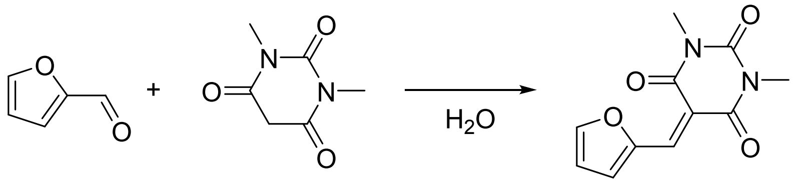

Reaction Yield	
**High School Students**	**Undergraduate Students (in pairs)**
69%	50%
59%	51%
65%	39%
72%	55%
70%	64%
52%	77%
66%	82%
**Average Yield**	
56%	60%

Compound characterization

5-(furan-2-ylmethylene)-1,3-dimethylpyrimidine-2,4,6(1H,3H,5H)-trione (BAF)

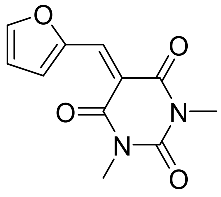

^{1}H NMR (300 MHz, $CDCl_3$) δ 8.64 (d, J = 3.8 Hz, 1H), 8.44 (s, 1H), 7.85 (d, J = 1.6 Hz, 1H), 6.74 (d, J = 2.7 Hz, 1H), 3.41 (s, 3H), 3.40 (s, 3H).

^{13}C NMR (75 MHz, $CDCl_3$) δ 162.6, 161.0, 151.5, 151.3, 150.6, 141.2, 128.2, 115.3, 111.5, 29.1, 28.4.

Sample	Melting point (ºC)
1	201-202
2	200-201
3	202
4	201
5	201-202
6	201-202
7	201

6-(furan-2-ylmethylene)-3,3-dimethylcyclohexane-1,2,4,5-tetraone (MAF)

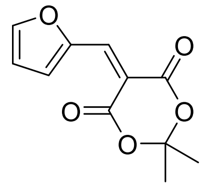

^{1}H NMR (300 MHz, $CDCl_3$) δ 8.46 (d, J = 3.8 Hz, 1H), 8.35 (s, 1H), 7.84 (d, J = 1.6 Hz, 1H), 6.75 (dt, J = 3.0, 0.9 Hz, 1H), 1.76 (s, 6H).

^{13}C NMR (75 MHz, $CDCl_3$) δ 163.4, 160.4, 150.6, 150.3, 141.4, 128.3, 115.4, 107.6, 104.7, 27.7.

Melting point = 87-91 ºC

^{1}H NMR Spectra (300 MHz, $CDCl_3$)

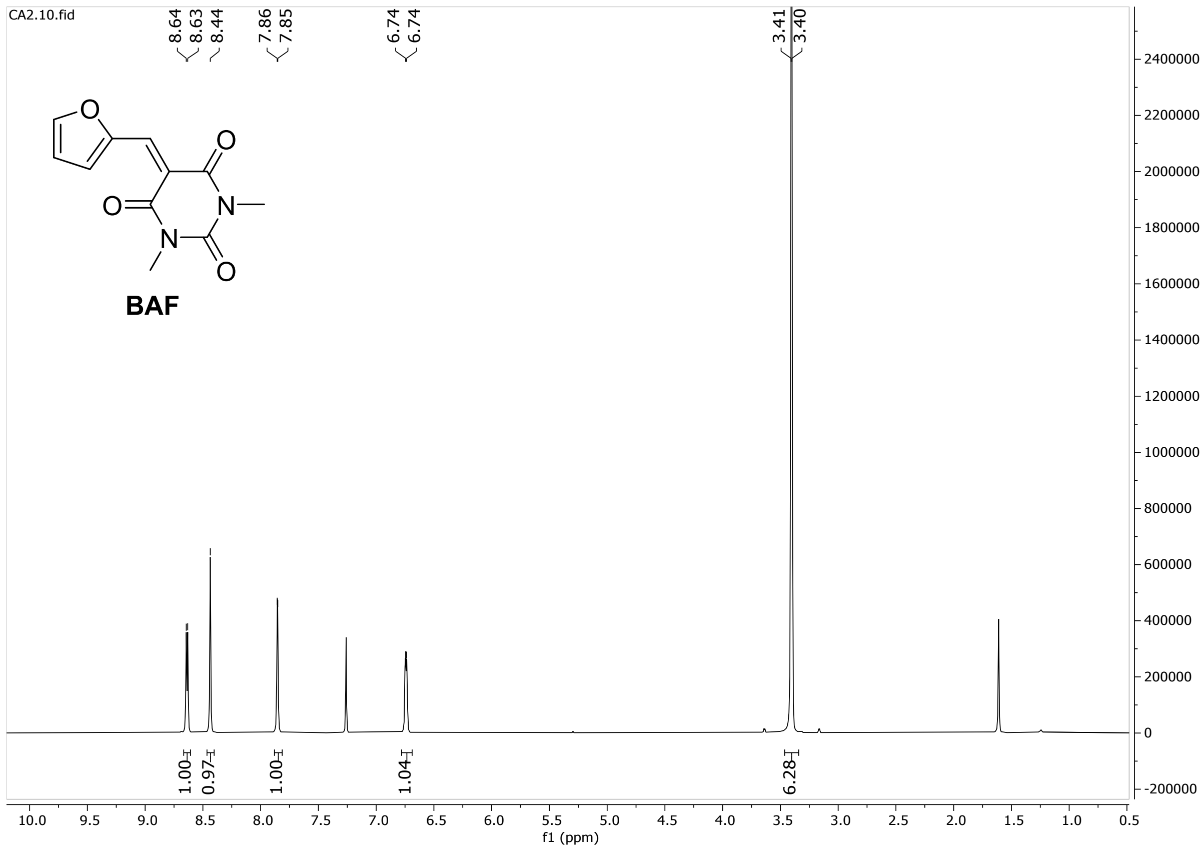

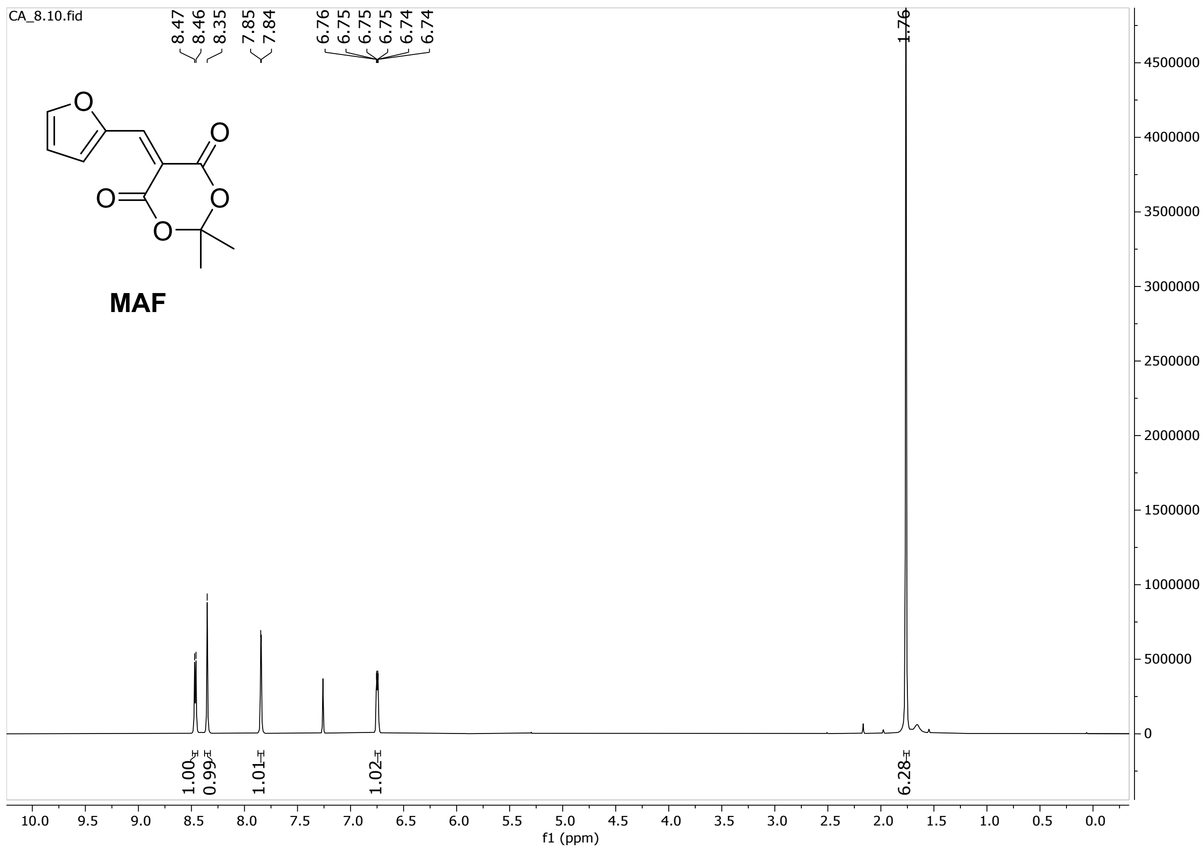

^{13}C NMR Spectra (75 MHz, $CDCl_3$)

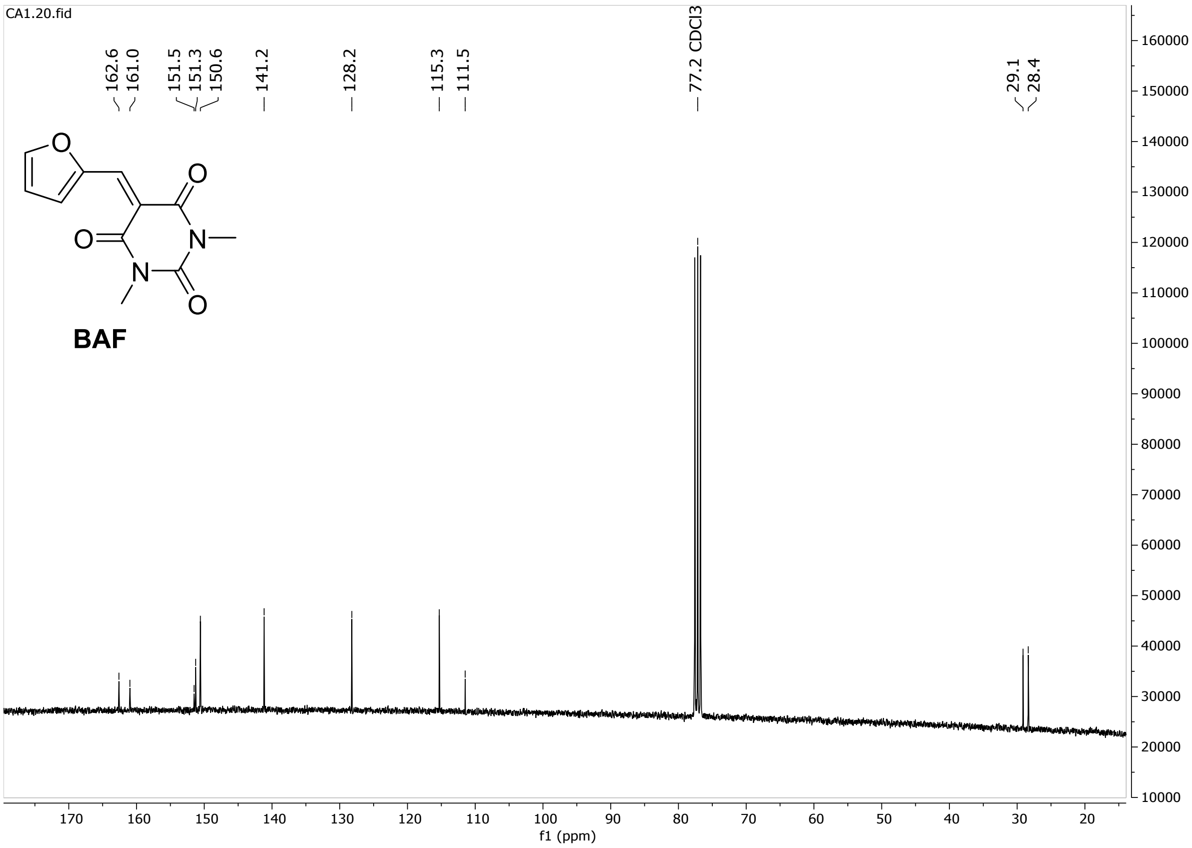

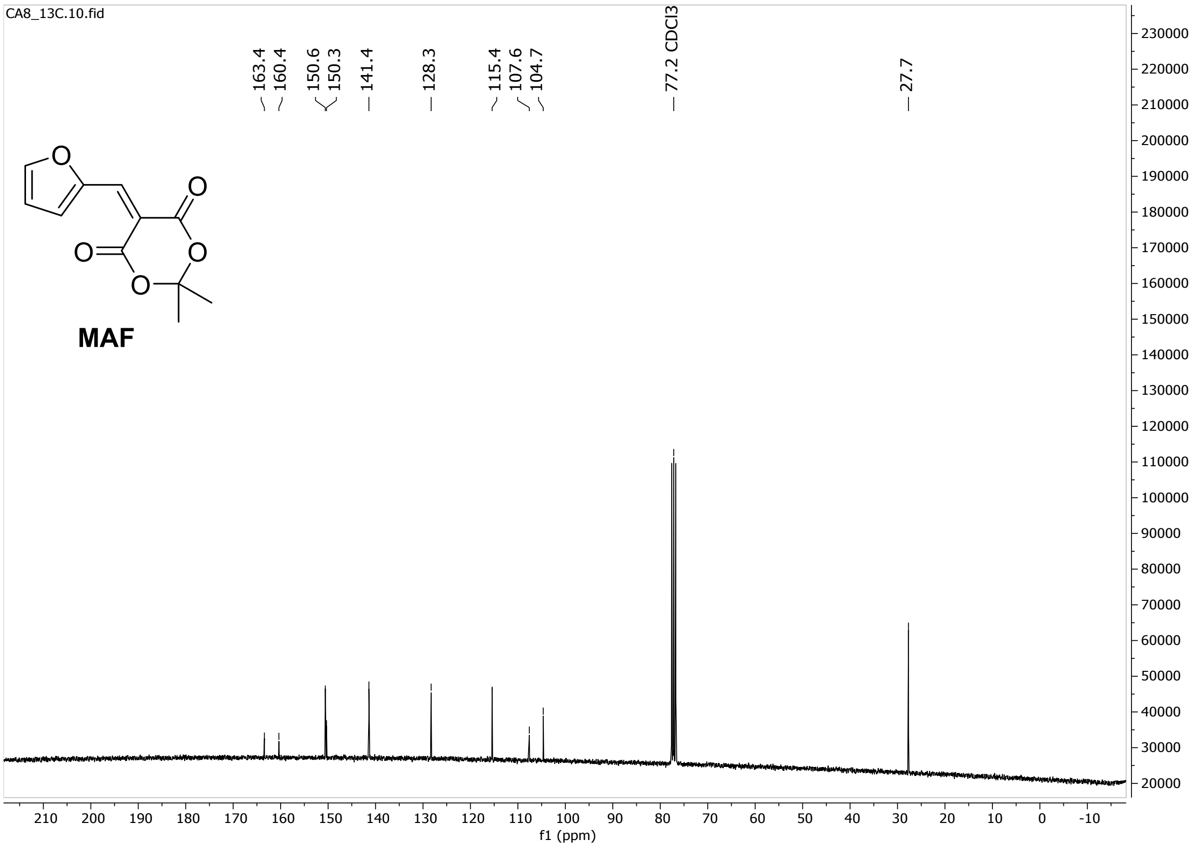

Thin Layer Chromatography

Group	Rf of amine 1-6					
	**1**	**2**	**3**	**4**	**5**	**6**
1	0.3	0.5	0.1	0.3	0.2	0
2	0.5	0.6	0	0.2	0.2	0
3	0.5	0.6	0.02	0.2	0.2	0
4	0.5	0.6	0.1	0.3	0.1	0
5	0.4	0.6	0	0.2	0.2	0
6	0.5	0.6	0.1	0.2	0.2	0
7	0.3	0.4	0	0.2	0.1	0

TLC examples from students (UV on top, stained on the bottom):

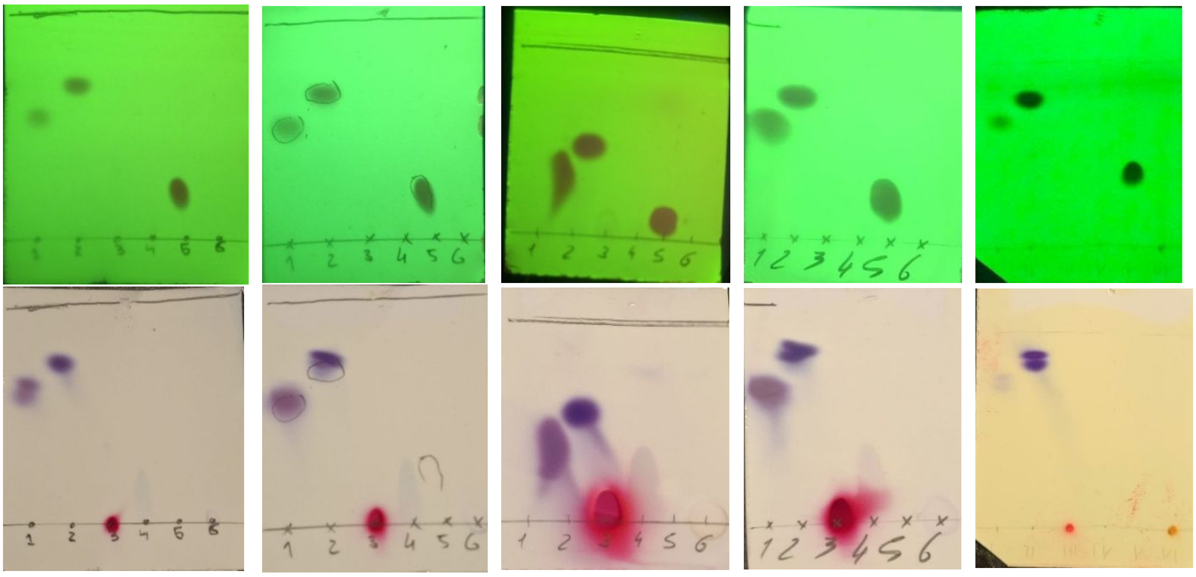

Student handouts

Preparation of An Amine Colorimetric Detector from Furfural

Theory

The nucleophilic addition of an amine (**1**) to a furfural Knoevenagel product (**2**), yields compound **3** after a ring opening reaction. Molecules such as **3** are highly coloured, due to an electron donor and acceptor units, connected through a π conjugated system.

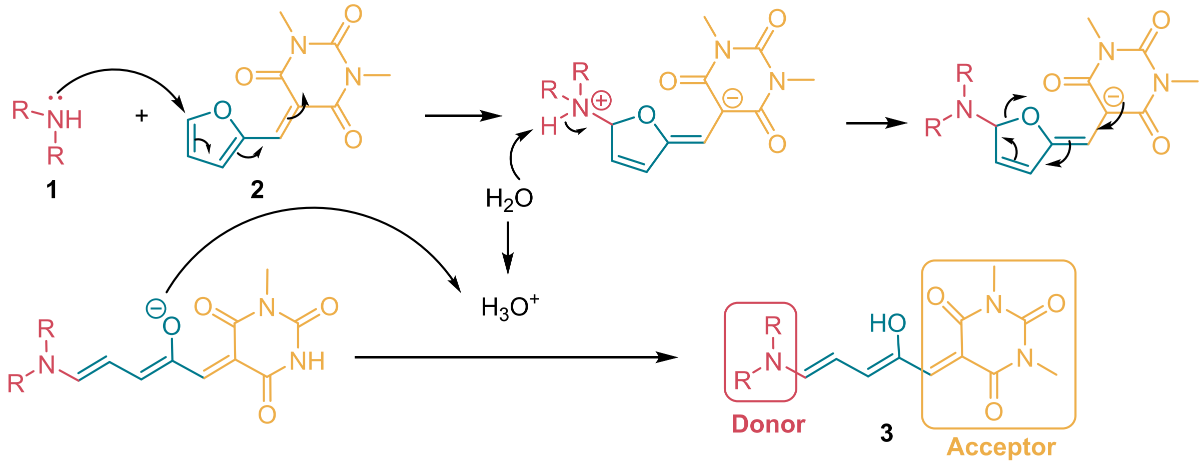

Molecules **2** can be used as a visual indicator of the presence of amines in solution, in which the fast formation of compound **3** and the appearance of strong coloration yields a positive result. Different strengths of electron donating capacity of each amine originates distinct hues of **3**, while their ability to act as nucleophiles in the first step of the reaction above dictates the appearance of colour.[1]

Green chemistry has emerged as a cornerstone for the development of more sustainable chemical processes, by extending the notion of synthetic efficiency beyond yield to include waste prevention, atom economy, safer reagents and solvents, lower energy input, and the preferential use of renewable feedstocks.[2,3] This is highlighted in the 12 principles of green chemistry described by Anastas and further expanded by Sheldon and others, such as 1. Waste prevention instead of remediation 2. Atom efficiency 3. Less hazardous/toxic chemicals 4. Safer products by design 5. Innocuous solvents and auxiliaries 6. Energy efficient by design 7. Preferably renewable raw materials 8. Shorter syntheses (avoid derivatization, protections and deprotections) 9. Catalytic rather than stoichiometric reagents 10. Design products for degradation 11. Analytical methodologies for pollution prevention 12. Inherently safer processes

In this context, sustainability can be viewed as the broader objective, whereas green chemistry provides the practical principles through which that objective can be pursued in chemical manufacturing.[3] The use of furfural as a substrate is particularly attractive from this perspective, since furfural is a biomass-derived platform molecule accessible from lignocellulosic feedstocks, thereby contributing to reduced reliance on fossil carbon and aligning with the principle of using renewable raw materials (7). Likewise,

the use of water as solvent is advantageous from a sustainability point of view, as it can replace more hazardous or environmentally burdensome organic solvents thus adhering to principles 3 and 5. As highlighted by Sheldon, the conversion of renewable feedstocks should ideally be coupled to environmentally friendly solvents, preferably water, together with efficient catalytic methodologies and low-waste process design. Accordingly, the combination of furfural as a renewable substrate and water as solvent represents a meaningful strategy toward more sustainable synthesis in accordance with the 12 principles of green chemistry.

Part I: Knoevenagel condensation

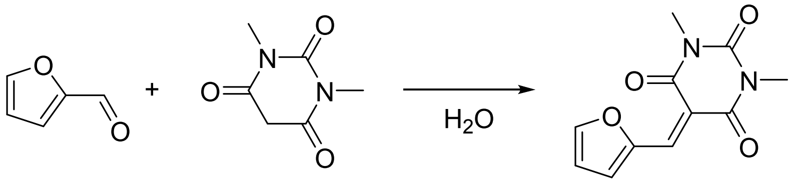

Furfural **Barbituric acid** **BAF (2)**

Synthesis:

1. In a 25 mL round bottom flask, add 276 mg of *N,N*-dimethylbarbituric acid (1.77 mmol, 1.08 equiv.).

2. Measure 13 mL of distilled water in a measuring cylinder and add it to the round bottom flask. Add a magnetic stirrer, cap the flask and place it over a stirring plate.

3. With a gastight syringe, add 136 µL (1.64 mmol, 1 equiv.) of furfural and place under strong stirring for 15 minutes.

4. Cool the reaction flask in an ice bath until formation of a precipitate.

5. Filter the precipitate in a Büchner funnel. Wash the solid with cold distilled water and transfer the solid to a 100 mL Erlenmeyer flask.

Purification:

6. Add ethanol to fully cover the solid and the Erlenmeyer flask's bottom. Place the flask over a heating plate.

7. Heat to flask until ethanol starts to boil. Slowly add more ethanol, allowing the solution to resume boiling, until full dissolution of the solid. (If there are any solids that do not dissolve in ethanol, filter the solution to a second hot Erlenmeyer flask, using a hot funnel and filter paper).

8. Let the solution slowly cool to room temperature and allow crystals to form.

9. Cool the solution in an ice bath for 5 minutes and filter the crystals through a Büchner funnel. Wash the crystals with cold ethanol.

10. Store the product in a weighted vial and leave it drying at 60ºC in an oven for 10 minutes.

Part II – Sample preparation of nitrogen bases:

Prepare six samples of nitrogen containing compounds in properly labelled test tubes. For liquid samples, use gastight syringes.

Sample 1: 20 µL of aniline in 2 mL of ethyl acetate.

Sample 2: 20 µL of 1,2,3,4-tetrahydroquinoline in 2 mL of ethyl acetate.

Sample 3: 20 µL of morpholine in 2 mL of ethyl acetate.

Sample 4: 20 µL of *N,N*-diisopropylethylamine in 2 mL of ethyl acetate.

Sample 5: 20 mg of acetanilide in 2 mL of ethyl acetate.

Sample 6: 20 µL of hexylamine in 2 mL of ethyl acetate.

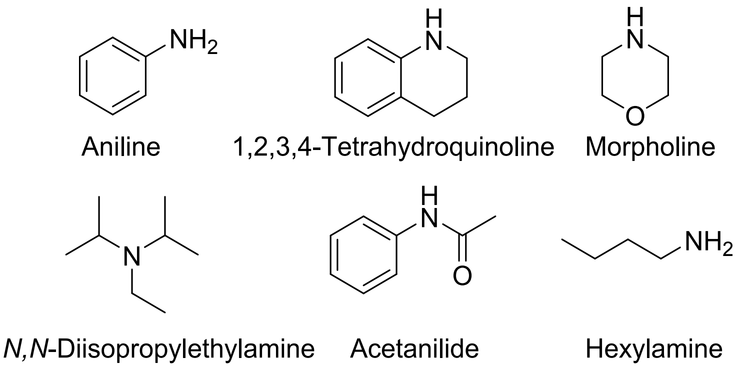

Part III – Amine detection:

1. In a 25 mL beaker, weight 45 mg of the product obtained in part I (**BAF**) and add 10 mL of ethyl acetate. Stir until full dissolution, obtaining the **staining solution**.

2. In a thin-layer chromatography (TLC) plate (Silica gel 60 F_{254}), add a drop of each sample prepared in part II with a disposable glass capillary.

3. Elute the TLC plate in a chamber with ethyl acetate/heptane (40:60).

4. After elution, dry the pate and check it under the UV lamp. Then, with a tweezer, submerge the TLC plate in the **staining solution** and remove it. Wait 5 minutes and note the results in the following table.

Sample	Nitrogen functional group	UV (254 nm) absorption	Color after staining	Rf
1		Yes □ No □		
2		Yes □ No □		
3		Yes □ No □		
4		Yes □ No □		
5		Yes □ No □		
6		Yes □ No □		

Objectives

1. Calculate the yield of Knoevenagel reaction between furfural and *N,N*-dimethylbarbituric acid.
2. Check the purity of BAF via measuring the melting point and comparing with reported values (201-202 ºC).
3. Fill out the table above with the observations from TLC analysis.

References

1. Rafael F. A. Gomes, Jaime A. S. Coelho, Carlos A. M. Afonso, "Synthesis and Applications of Stenhouse Salts and Derivatives", *Chemistry – a Europ. Journ.,* **2018**, *24*, 37, 9170-9186.

Questions and discussion

Question 1. In compound **2**, why does the amine attack the position 5 of the furan ring?

Question 2. What makes barbituric acid acidic? Justify presenting the resonance structures of its conjugated base.

Question 3. Why does acetamide not present any colour in the presence of the prepared stain?

Question 4. Sort all the nitrogen bases by ascending basicity.

Question 5. The two following spectra correspond to furfural and product **BAF**. Match the structures to their corresponding spectra and attribute each signal to the corresponding proton.

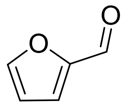

Furfural

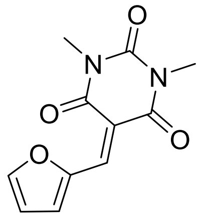

BAF

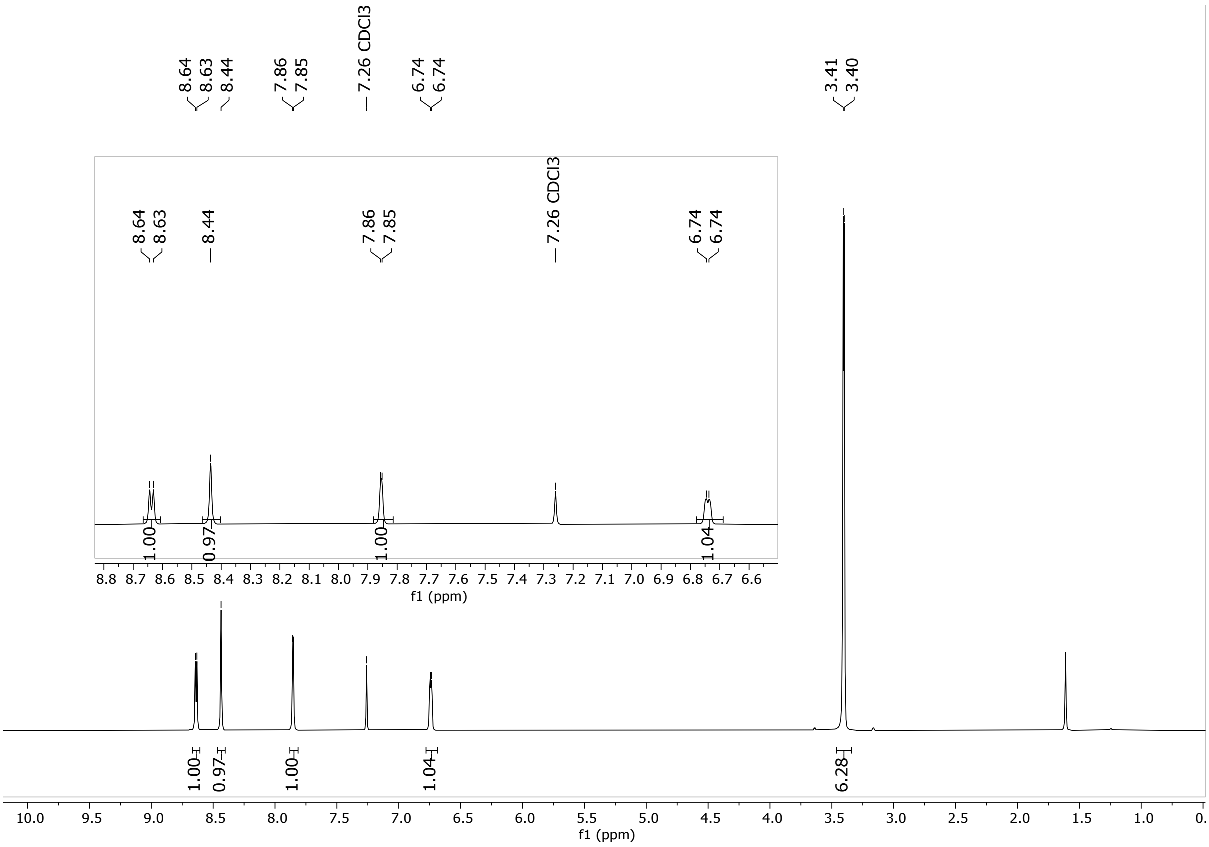
8.64
8.63
8.44
7.86
7.85
7.26 CDCl3
6.74
6.74
3.41
3.40
1.00
0.97
1.00
1.04
6.28
f1 (ppm)

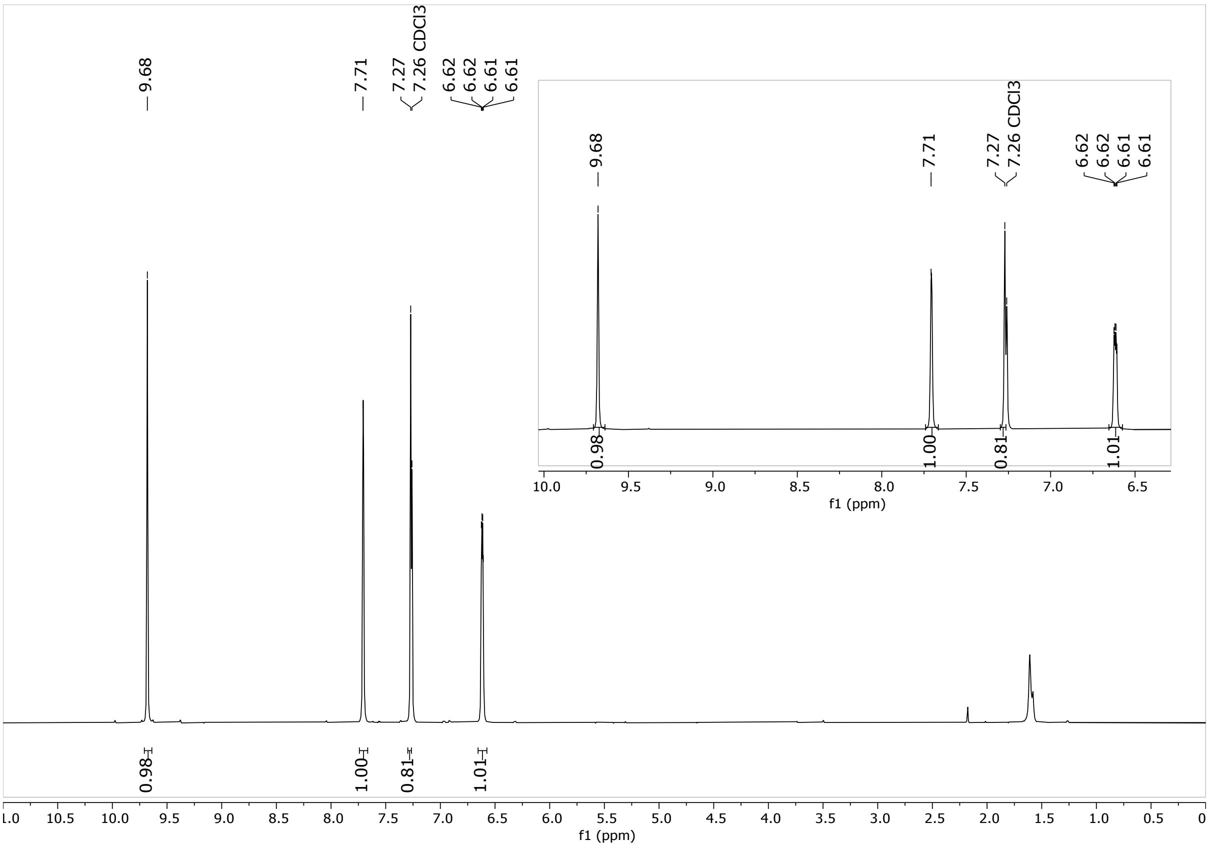
9.68
7.71
7.27
7.26 CDCl3
6.62
6.62
6.61
6.61
0.98
1.00
0.81
1.01
f1 (ppm)

Questions and discussion (solved)

Question 1. In compound **2**, why does the amine attack the position 5 of the furan ring?

Model Answer: Both positions 3 and 5 are the most electron deficient positions in the furan ring due to the influence of the electro-withdrawing barbituric acid substituent. If the addition occurs in position 3, the reversibility of the addition together with an unproductive pathway leads to no reaction. However, if the addition occurs in position 5, not only is the intermediary stabilized by more resonance structures, it can lead to a productive furan ring opening.

Question 2. What makes barbituric acid acidic? Justify presenting the resonance structures of its conjugated base.

Answer: The H between the two carbonyls is highly acidic due to the electron withdrawing effect of the carbonyls, plus the resonance of the negative charge between these carbonyls which in turn stabilizes the conjugated base.

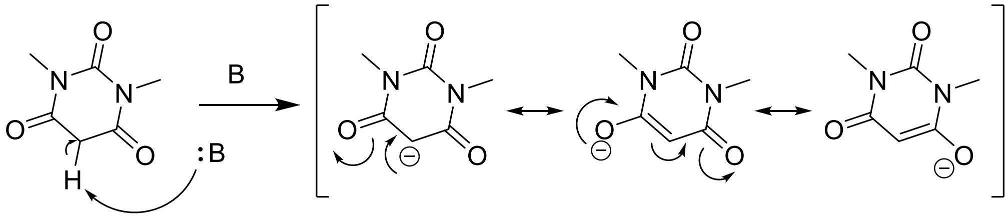

Question 3. Why does acetamide not present any colour in the presence of the prepared stain?

Answer: The furan ring opening that leads to the DASA only occurs upon the addition of a nucleophilic amine. Amides are not nucleophilic enough to perform this addition.

Question 4. Sort all the nitrogen bases by ascending basicity.

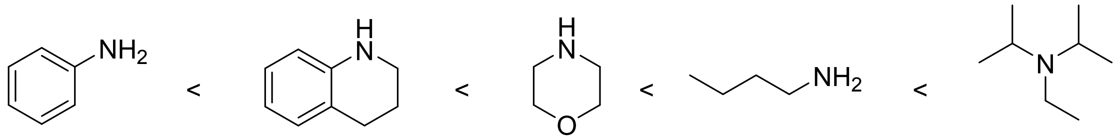

Aniline	1,2,3,4-Tetrahydroquinoline	Morpholine	Hexylamine	*N,N*-Diisopropylethylamine
pKa = 4.6	pKa = 5.1	pKa = 8.36	pKa = 10.64	pKa = 11.4

Question 5. The two following spectra correspond to furfural and product **BAF**. Match the structures to their corresponding spectra and attribute each signal to the corresponding proton.

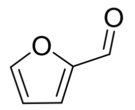

Furfural

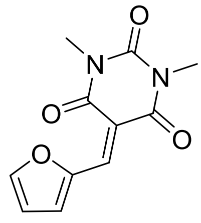

BAF

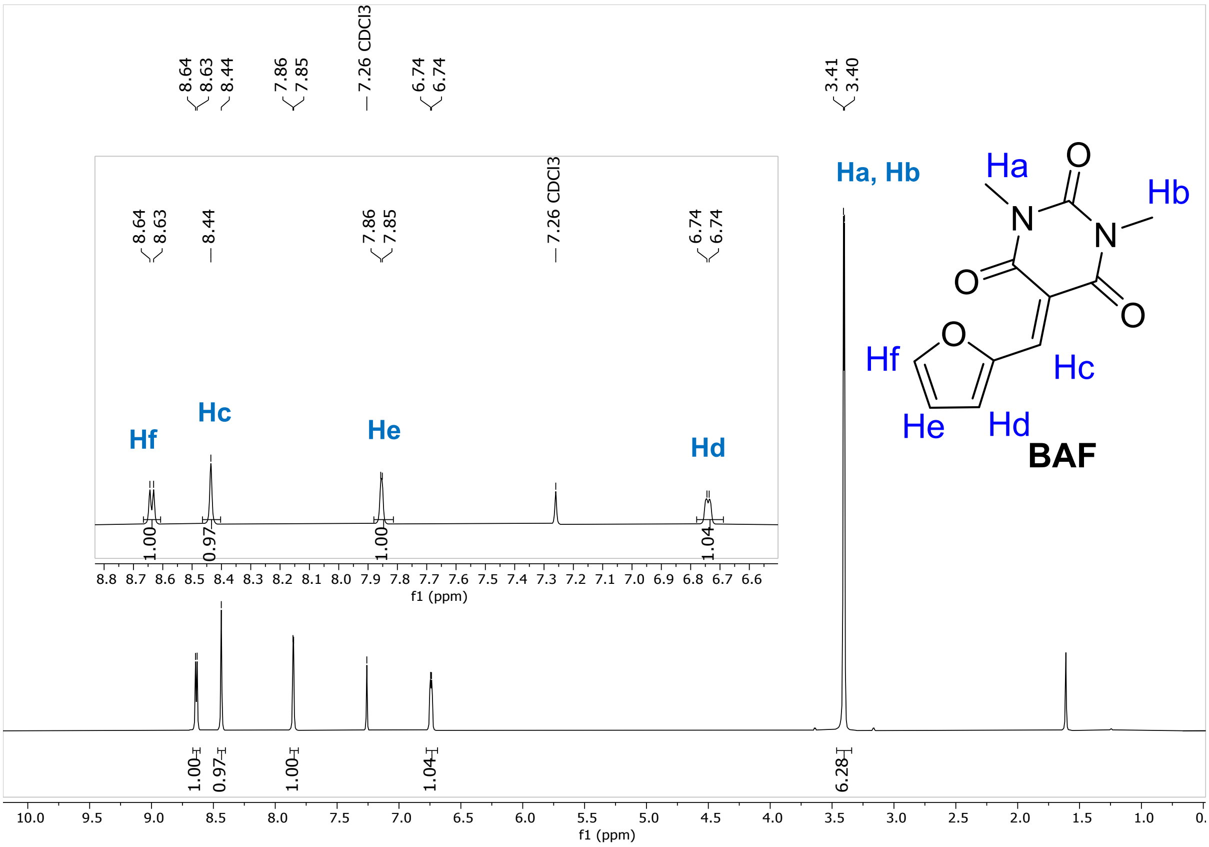
Ha, Hb
Ha
Hb
Hf
Hc
He
Hd
BAF
f1 (ppm)

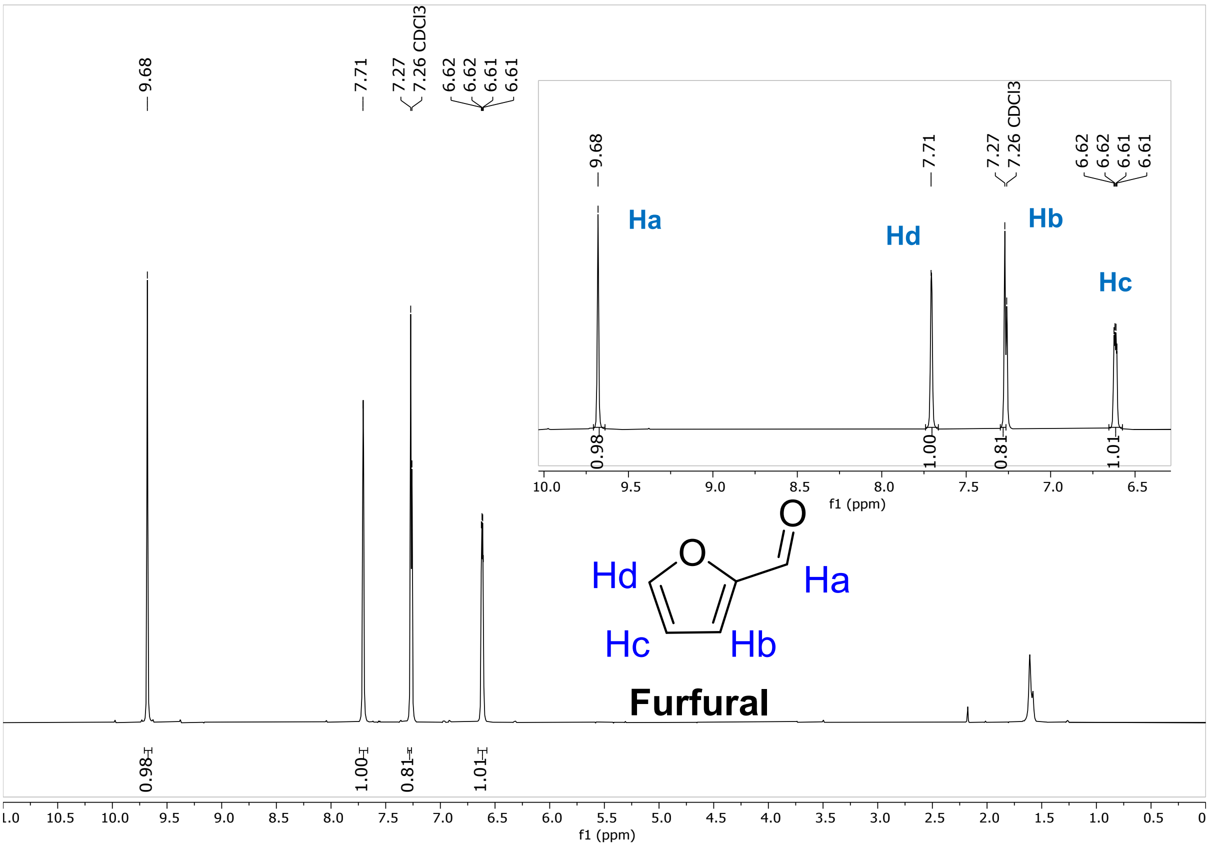
Ha
Hd
Hb
Hc
Furfural
f1 (ppm)
